# Extreme coastal erosion enhanced by anomalous extratropical storm wave direction

**DOI:** 10.1038/s41598-017-05792-1

**Published:** 2017-07-20

**Authors:** Mitchell D. Harley, Ian L. Turner, Michael A. Kinsela, Jason H. Middleton, Peter J. Mumford, Kristen D. Splinter, Matthew S. Phillips, Joshua A. Simmons, David J. Hanslow, Andrew D. Short

**Affiliations:** 1 Water Research Laboratory, School of Civil and Environmental Engineering, UNSW Sydney, 110 King Street, Manly Vale, New South Wales 2093 Australia; 2 Office of Environment and Heritage, NSW Government, 59 Goulburn Street, Sydney, New South Wales 2000 Australia; 3 School of Aviation, UNSW Sydney, New South Wales, 2052 Australia; 4 School of Geosciences, University of Sydney, Sydney, New South Wales 2006 Australia

## Abstract

Extratropical cyclones (ETCs) are the primary driver of large-scale episodic beach erosion along coastlines in temperate regions. However, key drivers of the magnitude and regional variability in rapid morphological changes caused by ETCs at the coast remain poorly understood. Here we analyze an unprecedented dataset of high-resolution regional-scale morphological response to an ETC that impacted southeast Australia, and evaluate the new observations within the context of an existing long-term coastal monitoring program. This ETC was characterized by moderate intensity (for this regional setting) deepwater wave heights, but an anomalous wave direction approximately 45 degrees more counter-clockwise than average. The magnitude of measured beach volume change was the largest in four decades at the long-term monitoring site and, at the regional scale, commensurate with that observed due to extreme North Atlantic hurricanes. Spatial variability in morphological response across the study region was predominantly controlled by alongshore gradients in storm wave energy flux and local coastline alignment relative to storm wave direction. We attribute the severity of coastal erosion observed due to this ETC primarily to its anomalous wave direction, and call for greater research on the impacts of changing storm wave directionality in addition to projected future changes in wave heights.

## Introduction

Extreme coastal storms such as hurricanes and extratropical cyclones (ETCs) can rapidly mobilise and redistribute vast quantities of sediment over large (>100 km) lengths of coastline, leading to erosion of beach and coastal dune systems^1^ and breaching or overtopping of coastal barriers^2, 3^. The result is damage to beach-front properties and infrastructure, flooding of coastal hinterland, and disturbance to beach environments and amenity. In a changing climate^4^, projected regional-scale changes in storminess^5, 6^ will possibly threaten the future resilience of many coastal communities worldwide^2^. At present, our ability to model and predict the damaging impacts of present and future extreme storms at the coast remains limited. A fundamental knowledge gap has been the paucity of regional-scale observational datasets of extreme beach erosion at sufficient spatial and temporal resolution, to adequately resolve the impacts of individual storms, and to capture the sensitivity of beach response to both regional- and local-scale alongshore complexity^7, 8^. Challenges include predicting the arrival of rapidly developing coastal storms (to ensure that the immediate pre-storm beach morphology is adequately quantified prior to the onset of storm conditions), as well as the practical difficulties of obtaining detailed measurements along large sections of coastline, within the short time-windows prior to and immediately following storm conditions.

Regional-scale observations of hurricane impacts along the U.S. east coast indicate that spatial variability in the morphological response corresponds strongly to local maxima in storm-induced total water levels (combing astronomical tide, storm surge, wave setup and wave run-up) relative to antecedent dune morphology^9^. While these dependencies provide a valuable framework for predicting hurricane impacts on low-lying coastlines^10^, the slower-moving and diffuse nature of ETCs means that coastal impacts during these events may be more a result of prolonged wave energy over several tidal cycles than the extreme and rapid increases in water levels observed during hurricanes^11, 12^. In this respect it is likely that a major control of the spatially-variable morphological response to ETCs along narrow continental shelf regions (where storm surges are significantly reduced) is the degree of exposure to storm wave energy. This is particularly the case for steep, embayed coastlines such as southeast Australia^13^, where prominent rocky headlands both attenuate and refract storm wave energy to create distinct localized gradients in wave exposure within embayed beaches in their lee^14^.

Here we present the first detailed measurements, both in terms of their immediacy pre- and post-storm as well as their high spatial resolution, of the regional-scale morphological response to an extreme ETC coastal erosion event. Referred to locally as an ‘east coast low’, storms of this genesis are the primary cause of coastal damage in this region and can produce long duration (i.e., days) near hurricane-force winds, intense precipitation and large ocean waves (up to 9 m deepwater significant wave height)^15, 16^. The direction of storm waves generated by ETCs in this region is typically from the south and south-east, depending on synoptic evolution^17^. Storm surge in comparison to other ETCs (e.g., North Atlantic nor’easters^12^) meanwhile is relatively small (maximum up to 0.7 m only), which is primarily due to the region’s steep and narrow continental shelf^18^. The June 2016 ETC was unusual in that an east coast low combined with a blocking high in the south Tasman Sea, which created a large (>2000 km) and relatively stable north-easterly fetch directed at the coastline for several days. While the recorded deepwater significant wave heights in the order of 6–7 m and surges of 0.2–0.5 m are considered moderate storm conditions for this coastline (equivalent only to annual recurrence intervals ~2–8 years^19^), the predominantly easterly wave direction for those conditions was unprecedented when compared to the available 25 years of directional wave measurements^20^. The damaging and widespread impacts of the June 2016 ETC event along the southeast Australia coastline were compounded by the storm’s coincidence with winter solstice spring tide, such that tidal levels over several days were up to 0.44 m higher than mean high water springs (mean spring tidal range = 1.25 m).

**Figure 1:**
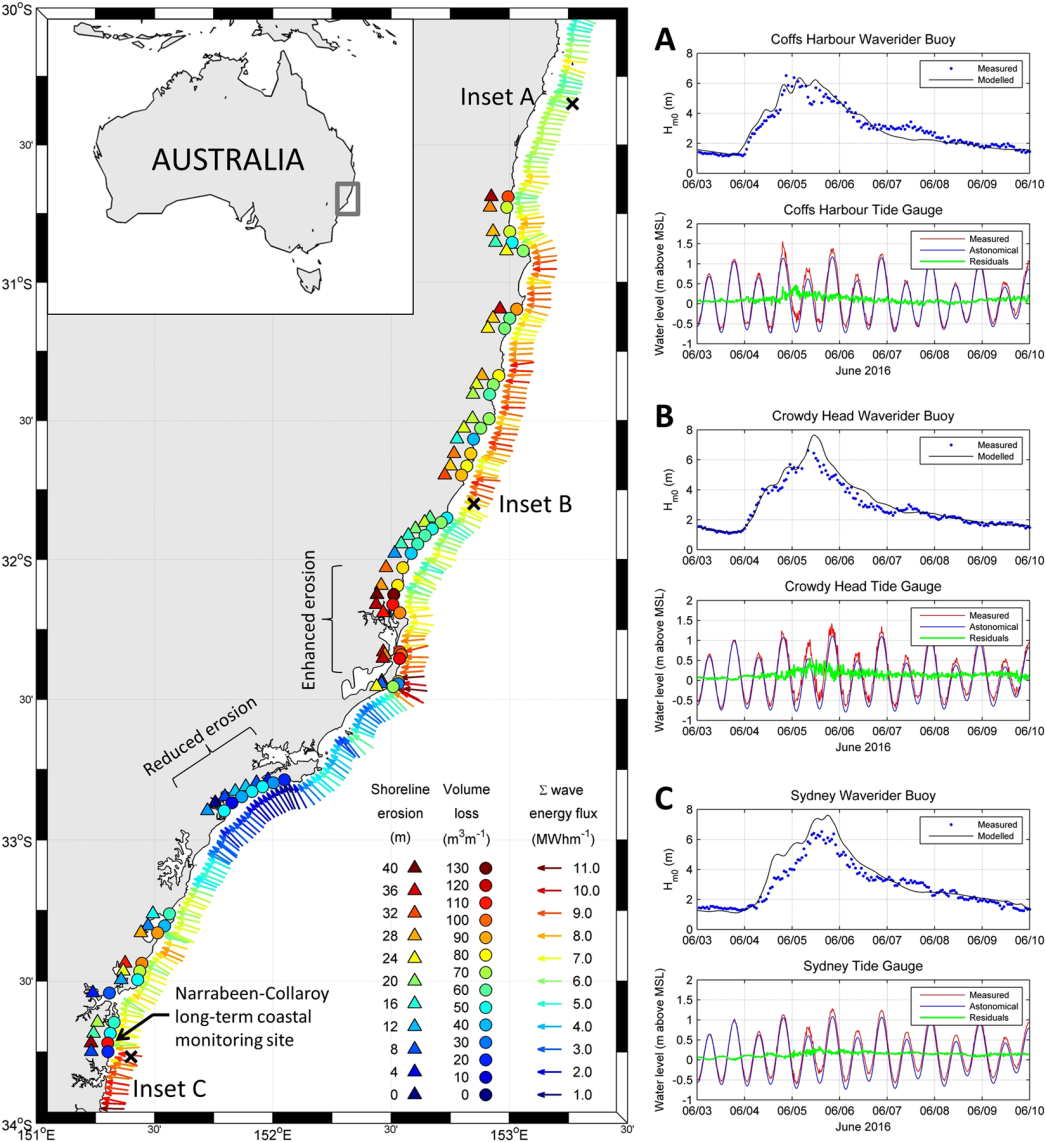
Regional-scale variability in morphological response to June 2016 storm controlled by exposure to anomalous storm wave direction. Enclosed triangles and circles denote alongshore-averaged shoreline erosion and alongshore-averaged sand volume loss above mean sea level measured by airborne Lidar collected pre and post-storm. Arrows represent the weighted-average direction and magnitude of the integrated storm wave energy flux during the storm event at the 30 m isobath, as derived from the high-resolution wave model. Insets A, B and C indicate comparisons between measured and modelled wave data and the associated measured water levels at three monitoring stations along the region. Map was created using the M_Map software package (version 1.4 h) for Matlab^47^.

High resolution topographic measurements of the morphological response to this storm were quantified by repeat airborne Lidar obtained immediately before and after the ETC (see Methods) along beaches extending from 30°40’S to 33°45’S (Fig. 1). The surveyed locations along this 400 km of coastline encompass a total of 177 km of sandy beaches, ranging in embayed beach length from 0.5 km to 21 km, and spanning 180° variation in local coastline orientation. Natural sandy beaches comprised 94% of the survey area and were characterised by wide sand berms (average width = 40 m) backed by vegetated foredunes. The remaining 6% of the survey area consisted of modified coastline, being adjacent to or within 500 m of existing coastal protection structures such as seawalls (4%) and breakwaters (2%). Morphological response was assessed at cross-shore transects spaced every 100 m along the survey region (1768 transects in total) by calculating, at each transect, the total subaerial sand volume change (i.e., all sand volume change above mean sea level) between the pre- and post-storm surveys, as well as the change in the shoreline position (defined by the mean high water contour).

## Results

### Regional and localized erosion

Based on the analysis, a total of 11.5 million m^3^ of sand was eroded from the subaerial beach (above mean sea level) over the full 177 km survey region, equating to average volume losses of 65 m^3^m^−1^ (i.e., on average 65 cubic meters of sand was eroded at every meter alongshore). The corresponding average shift in shoreline position was landwards by 22 m. Interestingly these average volume losses are similar in magnitude to those observed along the U.S. east coast in response to the 2012 Hurricane Sandy event^21^, highlighting the comparable morphological change that can occur during these less documented ETCs. The subaerial beach response across the entire survey region was overwhelmingly erosional (97% of all transects), with sediment transferred predominantly offshore to the subtidal zone, rather than redistributed alongshore. This onshore-offshore transfer of sand is further confirmed at the long-term coastal monitoring site, the 3.6 km Narrabeen-Collaroy embayment located within Metropolitan Sydney (refer Fig. 1). There, immediately pre- and post-storm detailed bathymetric measurements spanning the surfzone and nearshore were also conducted (see Methods), revealing a distinct deposition region in water depths between −2.5 m and −9.0 m of equivalent volume to the measured erosion above mean sea level (Supplementary Fig. S1).

To examine spatial variability in morphological response to the ETC at the regional scale, volume and shoreline changes were averaged alongshore within each embayed beach compartment defined by prominent rocky headlands, or for longer embayments, to a maximum alongshore length of 5 km. This alongshore-averaging resulted in a total of 52 individual coastal sectors for further regional-scale analysis. Figure 1 indicates that at this regional scale, the magnitude of subaerial volume loss for each sector (enclosed circles, Fig. 1) varied from 12 m^3^m^−1^ to 131 m^3^m^−1^ and shoreline change from 0 m (i.e., no change) to 40 m of landward recession. A distinct sub-region of enhanced subaerial sand volume loss is evident in the central survey region between 32°07’S and 32°21’S, which is contrasted by an area of reduced volume loss immediately to the south (between 32°47′S and 32°52′S, refer Fig. 1). High-resolution and regional-scale modelling of nearshore wave conditions throughout the survey area during the storm event (see Methods) indicates that these distinct differences in the observed subaerial response correspond to equivalent variations in storm wave exposure (represented at this regional scale by the integrated wave energy flux towards the coast at the 30 m isobath, ∑P_x,30_). We attribute this to the broad scale shift in coastline orientation from easterly to more southerly at this location (refer Fig. 1). Across the entire surveyed region, spatial variability in subaerial volume loss at the regional scale (i.e., average sand losses above mean sea level for each coastal sector) is significantly controlled by storm wave exposure, ∑P_x,30_ (r = 0.52, N = 52, p-value < 0.0005).

**Figure 2:**
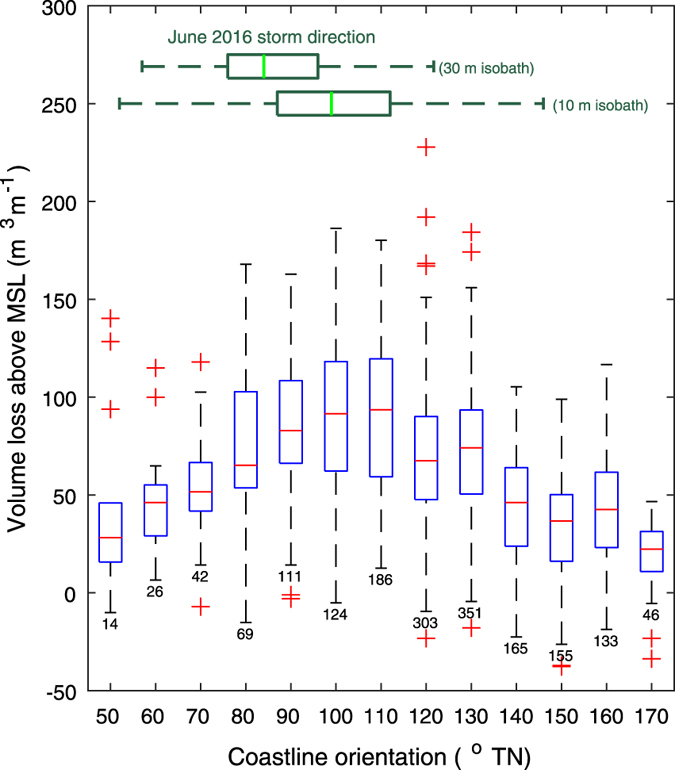
Localized sand volume change due to storm increases approximately three-fold for coastline locations in direct exposure to storm wave direction. Blue box-plots indicate the interquartile range and median (red line) of measured sand volume change above mean sea level (MSL) for transects sorted into 10° coastline orientation bins relative to true north. Associated whiskers and numbers denote the data range and number of transects respectively. Outliers are shown with red plus symbols and represent data 1.5 x the interquartile range. The range in weighted-average storm wave directions along the survey region at the 30 m and 10 m isobaths are indicated by the green box-and-whisker plots.

Considering both regional-scale and more localized (i.e., variation along individual embayed beaches) coastline exposure to storm wave energy fluxes, Fig. 2 indicates the variability in subaerial volume loss for all 1768 individual survey transects relative to the localized coastline orientation (grouped into 10 degree coastline orientation bins). The range of wave directions based on the modelled nearshore wave conditions is also indicated, with wave model output at both the 30 m and 10 m isobaths presented to capture the shift and spread in predominant direction due to wave transformations across the inner shelf. The results show an approximately three-fold increase in the median subaerial volume loss for individual survey transects directly aligned (coastline orientation ≈ 90–110° TN) to the predominantly easterly direction of the storm when compared to more northerly-oriented (coastline orientation ≈ 50° TN) and more southerly-oriented (coastline orientation ≈ 170° TN) transects. The general reduction in subaerial volume loss for these more northerly and southerly-oriented transects is attributed to the additional wave attenuation both at the regional scale by extended passage across the continental shelf and at the local scale by rocky headlands at beach extremities. The importance of localized inshore storm wave conditions to the measured erosion response is further demonstrated by analysing the localized storm wave exposure along the survey region, represented at the local scale by the integrated wave energy flux towards the coast at the 10 m isobath, ∑P_x,10_. A significant correlation is observed between subaerial volume losses and ∑P_x,10_ for all individual survey transects (r = 0.31, N = 1768, p-value < 0.00001), although at a reduced magnitude compared to the regional-scale analysis (r = 0.52). This reduced positive correlation between subaerial volume losses and storm wave exposure at the local scale (compared to the alongshore-averaged regional scale analysis) is due to increased variability between volume losses at the 100 m-spacing of individual transects (see Supplementary Data S1 to download and browse a Google Earth KML file of the entire subaerial volume change dataset). Close inspection of the locations of extreme individual subaerial volume losses (which reach a maximum of 228 m^3^m^−1^) reveals highly localized effects at specific transects, such as those in close proximity to coastal structures or complex offshore bathymetry; both features that are commonly associated with the formation of localized and highly-erosive megarips^22^.

### Historical perspective

**Figure 3:**
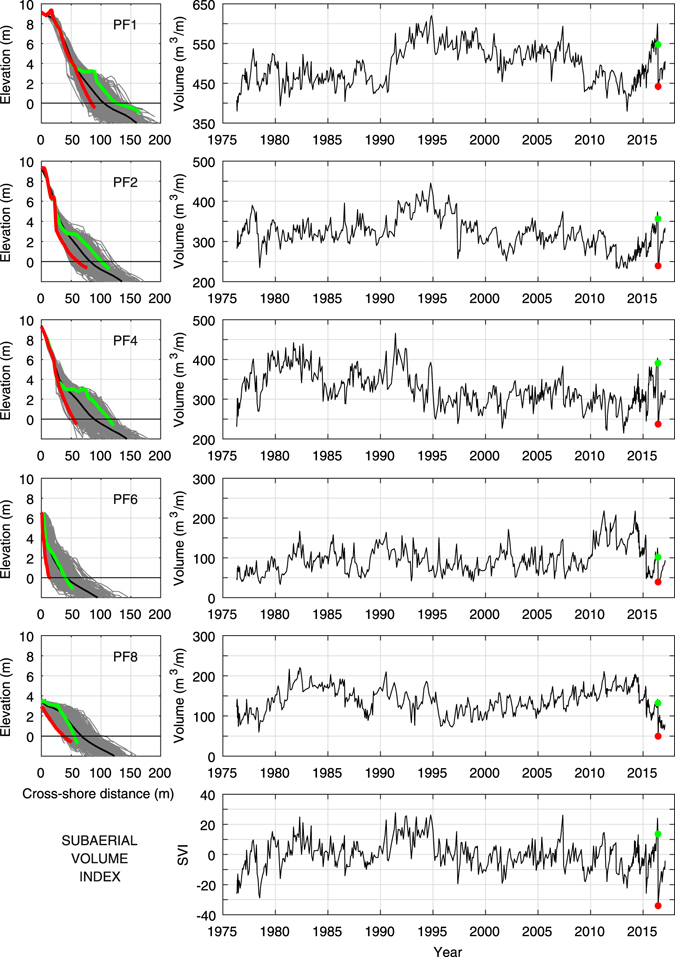
Rapid beach volume change due to June 2016 storm compared to four decades of subaerial beach variability. Data is from the Narrabeen-Collaroy long-term coastal monitoring site, where five transects (PF1, PF2, PF4, PF6 and PF8) have been monitored monthly between 1976 and 2016. Left panels indicate transect cross-sections over the entire forty-year measurement record, with the mean cross-section shown as a black line and the immediately pre and post-storm cross-sections as green and red lines respectively. Right panels indicate the associated time-series of subaerial beach sand volumes above mean sea level as well as the Subaerial Volume Index (SVI), a measure of the overall subaerial volume relative to the long-term average. Green and red dots denote the immediately pre and post-storm values respectively of sand volumes and SVI.

Placing these recent observations within their historical context, we compared subaerial volume loss measurements for the June 2016 ETC with those of previous storms at the Narrabeen-Collaroy long-term coastal monitoring site, where five survey transects spaced along the embayed beach have been surveyed on a monthly basis since 1976^23^. Using the average subaerial volume loss across all five transects between surveys as a gauge of storm severity, we find that the June 2016 ETC was the most severe erosion event measured in the last four decades and 36% greater than the secondmost erosive event (Table 1). Figure 3 illustrates the June 2016 beach volume change at these five transects relative to the past 40 years of recorded subaerial beach variability. The Subaerial Volume Index (SVI), a measure of the overall (i.e., integrated across all five survey transects) subaerial beach volume relative to the long-term average^24^, is also indicated in Fig. 3 (bottom panel). Immediately prior to the onset of the storm the subaerial beach (green profiles and dots, Fig. 3) was in a strongly accreted state relative to the long-term average (SVI = +13.6, equivalent to the 94^th^ percentile of all SVI measurements). Just four days later and immediately following the storm (red profiles and dots, Fig. 3), the subaerial beach was in its most depleted state since measurements began in the mid 1970s (SVI = −34.0). We attribute the severity in beach volume change observed during this event primarily to the anomalous easterly direction of the storm waves, which exposed the entire embayment (including the usually-sheltered southern section of the embayment) to enhanced storm wave energy fluxes inshore of the embayment headlands. Past south and south-easterly storms of much greater intensity in terms of peak significant wave height and integrated deepwater wave energy flux (e.g., the August 1986 and May 1997 storms, Table 1), as well as more intense storms at similar peak water levels (e.g., April 2015, Table 1), resulted in significantly less subaerial beach volume change, due to the sheltering influence of the southern headland.

**Table 1:** The June 2016 storm ranked as the most erosive storm event over the last forty years (1976–2016) at the Narrabeen-Collaroy long-term coastal monitoring site.

Rank	Storm	Peak H_m0_ (m)	Duration (hours)	Average wave direction (° TN)	Integrated deepwater wave energy flux (MWhm^−1^)	Peak water level (m)	Average volume change (m^3^m^−1^)
1	June 2016	6.5	74	106	9.7	1.29	103
2	May 1997	8.4	96	152	15.0	1.11	76
3	June 2007	6.9	65	149	9.5	0.95	73
4	April 2015	8.1	72	160	12.8	1.22	62
5	August 1986	7.2	108	136	21.3	1.06	58

Ranking is based on sand volume change above mean sea level averaged across the five survey transects at this site. Measured wave data are from the Sydney Waverider buoy and (August 1986 storm only) Botany Bay Waverider buoy. The average wave direction for the August 1986 storm was calculated from ERA-Interim wave reanalyses. Tide data are from the Sydney HMAS Penguin tide gauge and (August 1986 storm only) Sydney Fort Denison tide gauge. Tide level data are referenced to mean sea level.

## Discussion

Beyond the widely acknowledged likelihood that rising sea levels will impact sandy coastlines globally^25^, in recent years there has been increased research on the coastal implications of climate change induced wave climate variability ^1, 5, 6, 17, 26–28^. Global projections of deepwater wave climate change by 2100 (relative to present conditions) indicate considerable regional variability close to the coast, both in terms of modal deepwater wave conditions^5, 27^ as well as extreme conditions associated with storms^6, 28^. In some locations, projected decreases in both modal and extreme deepwater wave energy fluxes close to the coast have been interpreted as a possible dampener to the adverse coastal effects of sea level rise, whereas the combination of increased deepwater wave energy fluxes and rising sea levels could significantly amplify the coastal risks caused by climate change^6^. These projected shifts are most pronounced in the Southern Hemisphere, associated with a strengthening of Southern Ocean westerlies and a southerly shift of Southern Hemisphere ETC storm tracks^5, 6^. Our analyses highlight the additional importance of storm wave directionality and its pivotal role in modulating the degree of wave exposure along the coastal boundary. As demonstrated by the June 2016 event in southeastern Australia, storm wave directions that significantly enhance the degree of wave energy reaching the coast can lead to a major increase in episodic beach erosion, even when the absolute storm wave intensities of the event are comparably moderate. On the other hand, storm wave directions that decrease the regional wave exposure (through additional wave attenuation associated with an extended passage across the continental shelf, or refraction around localized non-erodible features) can lead to a significant decrease in the magnitude of episodic beach erosion.

The longer-term resilience of sandy beach systems to shifts in storm wave directionality is also likely dependent on the recurrence interval of such events. For low-frequency events characterised by an anomalous storm wave direction against a unimodal storm wave climate, beach resilence along modally more-sheltered coastal sectors may be reduced relative to typical storm directions and more-exposed locations. This is because, when storm conditions subside and the wave direction rotates back to more modal conditions, subtidal storm deposits in these locations can remain stranded, as wave energy necessary to return sediment to the beachface subsequently is reduced^14, 29^. The result is slower rates of beach recovery following the event and more prolonged periods of eroded conditions^30^. However on inter-annual time-scales, it is observed that shifts in storm wave directionality can occur in connection with climate oscillations such as the El Niño Southern Oscillation^31, 32^ and the North Atlantic Oscillation^33, 34^. Such shifts in storm wave directionality over these longer time-scales can result in a regional-scale adjustment in mophodynamic behaviour^26, 33, 35^, including rotation of the beach planform at the embayment scale^14^.

The morphological response to the ETC that impacted the southeast coastline of Australia in June 2016 was strongly characterised by sediment transport in the cross-shore direction, as evident by the great majority of transects in the survey region exhibiting substantial subaerial volume losses, and as shown by the accumulation of sediment in the surf zone and nearshore at the co-located long-term monitoring site. We attribute this dominance of cross-shore response primarily to the swash-aligned nature of this coastal region^22, 36^ as well as the predominantly shore-normal alignment of storm waves of this particular event (refer Fig. 2). Crucially, changing storm wave directionality can also significantly modify alongshore currents, driving an alongshore redistribution of beach sediments across regional coastlines that can influence coastal evolution over time-scales of years to decades^1, 17, 37^. Previous, as well as the present work, point to the necessity of regional downscaling of global wave climate projections, in order to gain a greater understanding of the directional sensitivity of wave transformation across continental shelves, to enable more reliable forecasts of future coastal response. Regarding episodic extreme beach erosion caused by ETCs, our results indicate that alongshore gradients in storm wave exposure at the coastal boundary provide a useful framework for estimating the corresponding regional-scale variability in subaerial beach volume losses.

## Methods

### Airborne Lidar surveys and data processing

Airborne Lidar data were obtained using a Riegl Q480i Lidar coupled with a NovAtel SPAN AG62 integrated Global Navigation Satellite System (GNSS) and inertial unit and flown using a twin-engine Piper PA44 airplane at an altitude of approximately 1000 ft (300 m). At this altitude, the data point density from the Lidar measurements is approximately 1 point every 1.6 m^2^. Previous comparisons using this system with concurrent *in situ* topographic surveys using RTK-GNSS mounted on an all-terrain vehicle (ATV)^38^ indicate a vertical accuracy of better than 0.2 m and horizontal accuracy of better than 0.5 m. The primary pre-storm Lidar flight encompassing 69% of the total 177 km of sandy coastline reported here was undertaken on 31 May 2016, five days prior to the onset of the storm. The additional survey data was derived from Lidar flights undertaken on 5 and 7 April 2016. Post-storm Lidar flights were undertaken over the two consecutive days 7 and 8 June 2016, less than 48 hours after the event had subsided.

Cross-shore transects for pre- and post-storm Lidar data processing were defined at 100 m intervals along embayed beaches of various degrees of planform curvature using a logarithmic spiral transform method^39^. Dune vegetation was removed from the data using a block-minimum and de-spiking algorithm^40^. Subaerial volume change *δV* was subsequently calculated at each transect using the equation:

1 $$\partial V={\int }_{{x}_{0}}^{{x}_{dc}}({z}_{1}-{z}_{2})dx$$

where x_0_ is the cross-shore position of the mean sea level contour, x_dc_ the cross-shore position of the dune crest where negligible change is observed and z_1_ and z_2_ are the pre- and post-storm elevation data respectively. Elevation data that did not intersect with mean sea level were extended down to this level using a linear model. The lower elevation bound of Lidar surveys were approximately 0.2 m above mean sea level for pre-storm surveys and 0.7 m above mean sea level for post-storm surveys.

### Regional wave model and spatial analysis

Wave conditions during the June 2016 ETC were simulated using the third generation spectral wind-wave model WAVEWATCH III^41^ forced with atmospheric conditions from the NOAA-NCEP Climate Forecast System Version 2^42^. The model included three nested regular grids – global (1° resolution), Australia (0.25° resolution), south-east Australia region (0.05°) – and an irregular mesh covering the study area, reaching 100 m resolution at 10 m water depth. Sensitivity testing of the model was carried out using Waverider buoy data for comparison, and the adopted configuration included ST2 source terms, JONSWAP bottom friction, and Battjes-Janssen depth-induced wave breaking^43^. The model performed well against data collected by three mid-shelf (70–80 m water depth) Waverider buoys deployed within the study area (Coffs Harbour, Crowdy Head and Sydney), accurately reflecting the timing, duration and intensity of storm wave conditions (Fig. 1, Insets A, B and C). The root mean square error of modelled significant wave heights over the duration of the ETC storm (3–10 June 2016) at these three buoy locations was 0.55 m.

To quantify both regional and local-scale variability in storm wave conditions at the coast, wave model time series data were analysed at 1 km resolution along the 30 m isobath (for regional-scale analysis) and 100 m resolution along the 10 m isobath (for local-scale analysis) over the duration of the ETC storm. The wave energy flux towards the coast, *P*_x,d_, was calculated along each isobath of depth *d*:

2 $${P}_{x,d}=nEC\,\cos \,\alpha $$

where *n* is a scaling factor $(n=\frac{1}{2}(1+\frac{4\pi d/L}{\sinh \,4\pi d/L}))$, *E* is the wave energy density $(E=\frac{1}{16}\rho g{{H}_{m0}}^{2})$, *C* is the wave celerity $(C=\frac{g{T}_{p}}{2\pi }\,\tanh \,\frac{2\pi d}{L})$, α is the angle between the wave direction and regional/local coastline orientation, *H*_*m0*_ is the significant wave height, *T*_p_ is the peak wave period and *L* is the wavelength.

Exposure to storm waves at each coastal sector (for regional-scale analysis) or individual survey transect (for local-scale analysis) was subsequently defined at the nearest model output location along isobath of depth *d* (*d* = 30 m and 10 m for regional and local-scale analysis, respectively) by integrating P_x,d_ over the duration of the storm event:

3 $$\sum {P}_{x,d}={\int }_{{t}_{1}}^{{t}_{2}}{P}_{x,d}dt$$

where *t*_*1*_ and *t*_*2*_ represent the up-crossing and down-crossing times respectively of the corresponding deepwater storm threshold (defined for this region^31^ as *H*_*m0*_ = 3 m). This storm exposure metric subsequently takes into account both the magnitude of wave energy fluxes for a certain water depth as well as the event duration and has been found to be an effective means of gauging the coastal response to storm events^44^.

### Narrabeen-Collaroy coastal monitoring site

Coastal monitoring at five transects along Narrabeen-Collaroy Beach commenced in April 1976 and has continued monthly up until the present date (November 2016). Between 1976 and 2006 these transects were surveyed using conventional survey techniques^45^. Since 2006 these surveys have been undertaken using RTK-GNSS^46^. All transects extend from a landward stable benchmark until at least mean sea level. For a complete description of coastal monitoring at this site, refer to ref. 21.

Pre- and post-storm bathymetric measurements for the June 2016 storm were undertaken using personal water craft (jetski) based eco soundings with RTK-GNSS positioning. The surveys were undertaken on 2 and 10 June 2016 and included 46 track lines spaced 50 m apart, with post-processing undertaken in Hypack.

## Electronic supplementary material

Supplementary Information
