# Supplementary material for: Extreme coastal erosion enhanced by anomalous extratropical storm wave direction
Source: Sci Rep. 2017 Jul 20;7:6033. doi: 10.1038/s41598-017-05792-1 (PMC5519658; doi:10.1038/s41598-017-05792-1)
Supplement: Supplementary file 1 — Supplementary Information [file 41598_2017_5792_MOESM1_ESM.pdf]

Extreme coastal erosion enhanced by anomalous extratropical storm wave direction

Mitchell D. Harley*[1], Ian L. Turner[1], Michael A. Kinsela[2], Jason H. Middleton[3], Peter J. Mumford[3], Kristen D. Splinter[1], Matthew S. Phillips[1], Joshua A. Simmons[1], David J. Hanslow[2], Andrew D. Short[4]

[1]Water Research Laboratory, School of Civil and Environmental Engineering, UNSW Sydney, 110 King Street, Manly Vale, New South Wales 2093, Australia, m.harley@unsw.edu.au

[2]Office of Environment and Heritage, NSW Government, 59 Goulburn Street, Sydney, New South Wales 2000, Australia

[3]School of Aviation, UNSW Sydney, New South Wales 2052, Australia

[4]School of Geosciences, University of Sydney, Sydney, New South Wales 2006, Australia

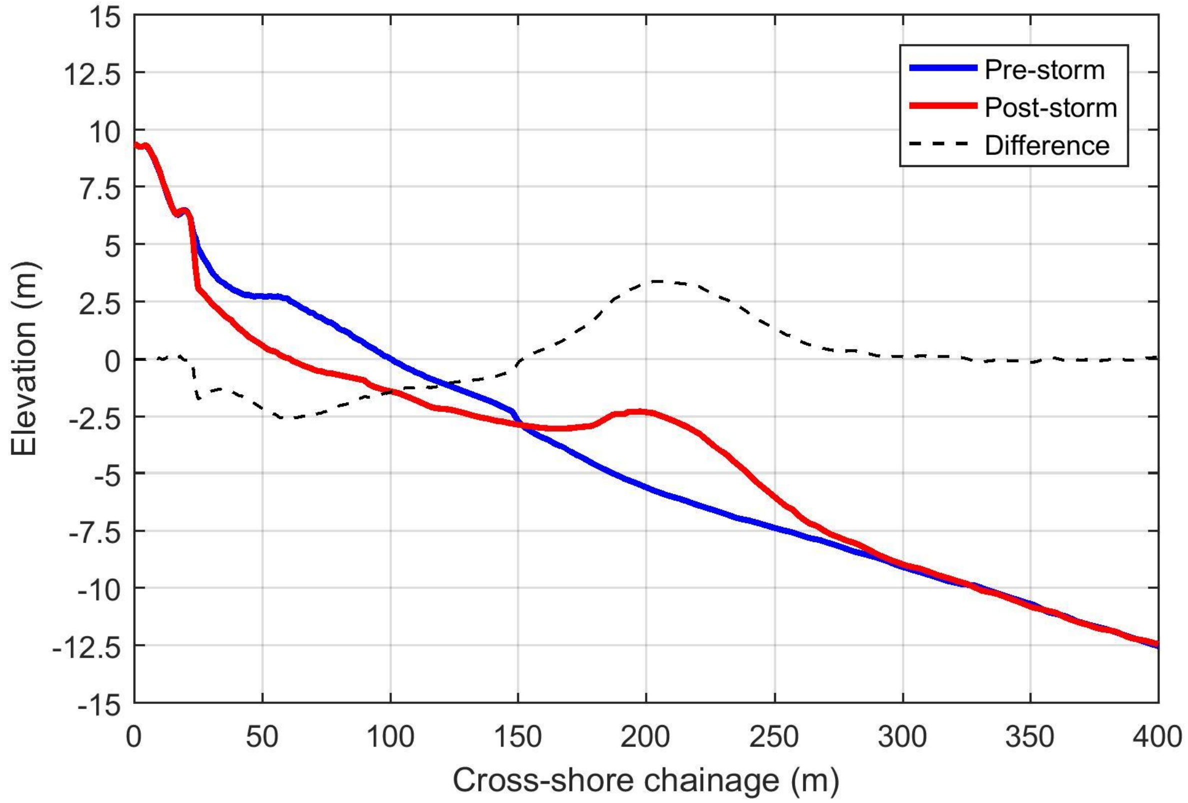

Supplementary Figure S1. Morphological change over complete beach profile due to June 2016 storm. Data is from the Narrabeen-Collaroy long-term coastal monitoring site (transect PF2), where immediate pre- and post-storm bathymetric data were collected in addition to the high-resolution regional topographic data.

Supplementary Data S1

Google Earth KML file of subaerial beach sand volume change due to the June 2016 east coast low storm in SE Australia can be downloaded via figshare repository here:

https://doi.org/10.6084/m9.figshare.4868219.v1

Use Google Earth software to browse dataset.
